# Notes on Genito-Urinary Therapeutics

**Published:** 1882-05

**Authors:** 


### NOTES ON GENITO-URINARY THERAPEUTICS.

ACONITE. Renal.—Sensitiveness of renal region, with shooting pains. Kidneys act slightly; urine has fragmentary casts and albumen stitches in kidneys and pressure in bladder, with retention or suppression of urine.

Vesical.—Inflammation of bladder, with constant urging; urine scanty, or passes in drops, burning and mixed with blood; tension, heat and tenderness over pubes; pinching around umbilicus; bladder feels painful when he walks.

Enuresis (*Bry.*, *Nit. ac.*, *Puls.*, *Rhus*), with thirst and diarrhœa (feces white). Must rise at night (12–3 A. M.) to urinate (pregnancy).

Diuresis, with headache or profuse sweat.

Retention from cold, especially in children, with much crying and restlessness.

Hæmaturia (*Canth.*, *Clem.*, *Lyc.*, *Merc. corr.*, *Ph. ac.*, *Puls. Sepia*, chiefly), with hæmorrhoids of bladder or anus, and burning distress in urethra.

Urinary Pains.—(Painful emission of bloody urine, by drops, *Canth.*, *Merc. corr.*)

Burning in urethra while urinating; at neck of bladder when not urinating.

Painful, anxious urging to urinate; frequent desire.

Violent burning in bladder.

Micturition difficult, painful and by drops (*Cann. ind.*, *Dig. Clem.*, *Mer. corr.*); faint feeling while urinating.

*The desire to urinate is accompanied* by great distress, *anxiety* and *restlessness.*

Character of Urine.—*Urine*, scanty, *red*, *hot*, *without sediment;* brown, burning, with brick-colored sediment.

Before urination, painful and anxious urging. Desire to urinate on touching the abdomen.

During urination, colicky pain (also, *Verat.*) Pain in glans penis; sensation of splashing of fluid in region of bladder; pinching about navel.

Sexual.—Desire increased; lasciviousness; amorous dreams; desire increased in the evening, with heat and sweat. Desire lessened; parts relaxed and tingling.

Involuntary emissions, even after coitus. Testicles feel hard and swollen, as if too full; bruised pain in scrotum (*Kalmia*); violent orchitis.

Crawling and tingling in scrotum (*Selen.*)

(Crawling and tingling in testicles, *Euphr.* and *Merc.*)

Children finger genitals (*Merc.*, *Zinc.*), with crying.

Scrotum drawn up; drawing pain in right testicle; left side of scrotum covered with vesicles, having a humid discharge.

Gonorrhœa with inflammation, burning and sudden, flying stitches, through glans penis.

Concomitants.—Fever (general dry heat, pulse *full and hard*), with great thirst and agonized tossing about; anxiety; fear of death; worse from exposure to dry, cold air.

AGARICUS. RENAL.—Pressing pain in region of kidneys, which disturbs sleep.

Stitches in right kidney.

Lameness in left kidney, sore to touch, with cramping pain extending into thighs. (*Kali c.?*)

VESICAL.—Paralytic weakness of sphincter vesical, can hardly retain urine.

Urine passes at intervals (flows and stops), dribbles away (*Bell.*, *Canth.*, *Caust.*, *Merc.*), penis being cold and shrunken.

Urine passes slowly in a small stream, or in drops; has to strain to increase the flow.

Urination with coldness down the legs, numbness and twitches.

Frequent desire to urinate; the quantity is very much increased even with diarrhœa.

Urination lessened, or profuse and colorless.

URINARY PAINS.—Cramp-like pain in groin during micturition.

Painful urging along the urethra; burning at orifice of urethra at night.

Sensation in urethra as though a drop of cold urine (*Nitric Acid*) was passing.

Sensation as if some urine remained in the urethra. (*Asper.*, *Ced.*, *Ery. a.*)

Stitches like a red-hot iron in the urethra.

Crawling, itching, tickling in orifice, ameliorated by cold water.

Crawling in forepart of penis, so bad he wants to squeeze the organ.

CHARACTER OF URINE.—Urine has a shining (iridizing Hg.) pellicle on surface. (*Natr. m.*, *Ph. ac.*)

Urine clear and watery; lemon-colored or bright yellow.

Urine dark yellow and hot.

Urine watery in forenoon; afternoon milky.

Sediment, red flocculent or powder-like; white sediment.

SEXUAL.—Great desire for embrace, penis relaxed; excited sexual desire, worse in mornings.

Sexual desire much diminished; nothing excites it.

Pollutions several nights during siesta (*Aloe*). After emissions, pains and weakness in thighs. Complaints from sexual excesses.

During coition, burning in urethra, most felt during ejaculation; emission of semen slight or late; voluptuous feeling deficient.

After coition, weakness, lassitude, loss of appetite, night-sweats; itching of skin at night.

Penis cold (*Ägar.*, *Cann.*, *Caps.*) and shrunken. (Glans penis cold, *Berberis*).

Drawing in testicles, with heaviness and discomfort.

Spasmodic drawing in left testicle and spermatic cord.

Excessive and painful retraction of testes toward inguinal rings; wants to press them off with his hands.

Left side of scrotum red and swollen; tickles and itches.

Excessive and unbearable itching of genitals.

CLINICAL.—Spermatorrhœa, with pains and weakness in thighs. Useful in long-running gleets, with above symptoms. For a yellow purulent gonorrhœal discharge; for a discharge of viscid, glutinous mucus from urethra.

